# Endoscopic bronchial occlusion for postoperative persistent bronchopleural fistula with computed tomography fluoroscopy guidance and virtual bronchoscopic navigation: A case report

**DOI:** 10.1097/MD.0000000000009921

**Published:** 2018-02-16

**Authors:** Masahiro Yanagiya, Jun Matsumoto, Masaaki Nagano, Masashi Kusakabe, Yoko Matsumoto, Ryutaro Furukawa, Sayaka Ohara, Kazuhiro Usui

**Affiliations:** a Department of General Thoracic Surgery, NTT Medical Center Tokyo; b Department of Thoracic Surgery, The University of Tokyo Graduate School of Medicine; c Department of Radiology; d Division of Respirology, NTT Medical Center Tokyo, Tokyo, Japan.

**Keywords:** bronchial occlusion, bronchopleural fistula, computed tomography fluoroscopy, virtual bronchoscopic navigation

## Abstract

**Rationale:**

The development of postoperative bronchopleural fistula (BPF) remains a challenge in thoracic surgery. We herein report a case of BPF successfully treated with endoscopic bronchial occlusion under computed tomography (CT) fluoroscopy and virtual bronchoscopic navigation (VBN).

**Patient concerns:**

A 63-year-old man underwent right upper lobectomy with concomitant S6a subsegmentectomy for lung adenocarcinoma. On postoperative day 24, he complained of shaking chills with high fever.

**Diagnoses:**

BPF with subsequent pneumonia and empyema.

**Interventions:**

Despite aggressive surgical interventions for the BPF, air leakage persisted postoperatively. On days 26 and 34 after the final operation, endobronchial occlusions were performed under CT fluoroscopy and VBN.

**Outcomes:**

The air leaks greatly decreased and the patient was discharged.

**Lessons:**

CT fluoroscopy and VBN can be useful techniques for endobronchial occlusion in the treatment of BPF.

## 1 Introduction

Postoperative bronchopleural fistula (BPF) is one of the most serious complications in thoracic surgery because it is strongly associated with fatal pneumonia and empyema.^[1,2]^ Prompt and appropriate management of BPF is necessary to improve the outcome of this potentially fatal clinical condition. Surgical interventions such as open window drainage and thoracoplasty are effective for control of infection due to BPF. Endoscopic fistula closure has also been reported as a favorable option for treatment of BPF.^[2]^

Although endoscopic bronchial fistula closure is a useful procedure for BPF, rapid identification of the affected bronchus is necessary to ensure the patient's safety and comfort. However, detection of BPF is often challenging because the initial bronchoscopy may fail to identify a small BPF.^[3]^

Computed tomography (CT) fluoroscopy guidance and virtual bronchoscopic navigation (VBN) can be useful methods for the diagnosis of peripheral pulmonary lesions because they allow for identification of the lesion location and the bronchi that reach the lesions.^[4,5]^ However, few reports have described the successful use of these techniques for bronchial fistula closure.^[6]^ We herein report a case of postoperative BPF with persistent air leakage that was successfully treated with endoscopic bronchial closure supported by CT fluoroscopy guidance and VBN. We obtained informed consent from the patient for reporting this case.

## 2 Case presentation

**Figure 1:**
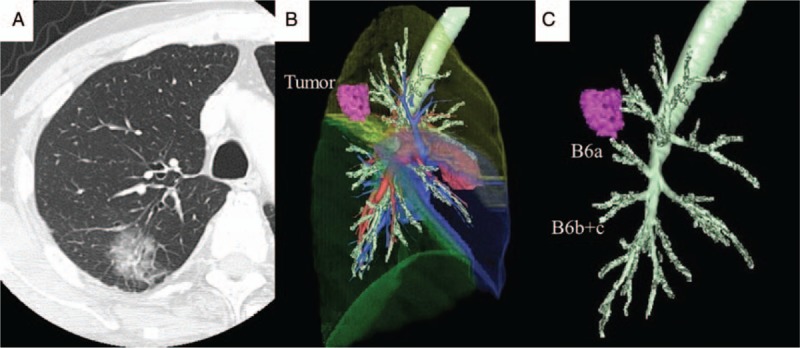
(A) Chest computed tomography (CT) showed a 31 mm diameter mass with ground-glass opacity in the dorsal segment (S2) of the right upper lobe. (B) Three-dimensional CT showed that the mass was located near the superior segment (S6) of the right lower lobe. (C) Three-dimensional CT also showed an anomalous B6a independently branching from the right lower lobe bronchus.

A 63-year-old man with a history of hypertension was referred to our department because of a chest radiograph abnormality that was incidentally found during a visit to a clinic for evaluation of coughing. Although his cough had improved, chest CT demonstrated a 31 mm diameter mass with ground-glass opacity that had increased in size within 1 month, located in the dorsal segment (S2) of the right upper lobe (Fig. 1A and B). The mass raised suspicion for primary lung cancer. Positron emission tomography showed no abnormal uptake. No detectable metastases were present in other organs. No biopsy was performed preoperatively because the mass was highly suspicious for lung cancer.

Chest CT also showed 2 stems of the superior segmental bronchus (B6) of the right lower lobe (Fig. 1C). The patient had anomalous B6a and A6a independently branching from the main stream. Because the nodule of S2 was located near S6a (Fig. 1B), we performed right upper lobectomy and concomitant right S6a subsegmentectomy with lymph node dissection.

**Figure 2:**
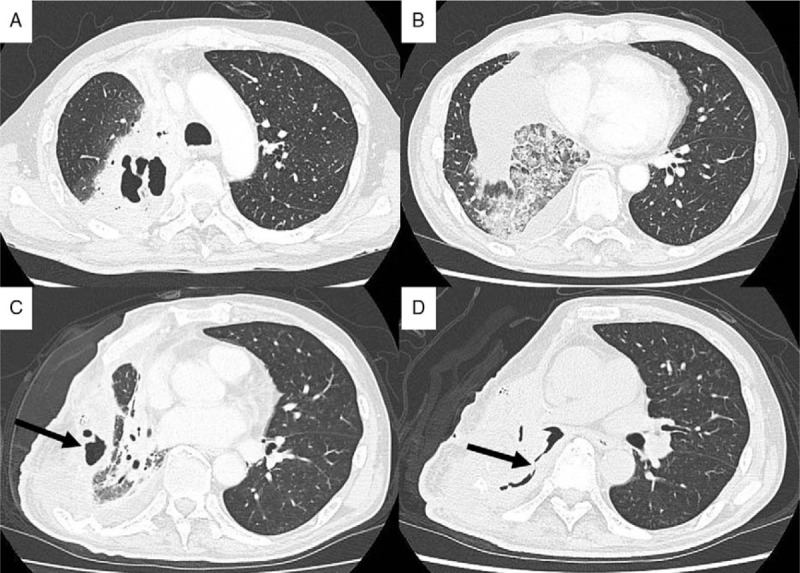
(A) Chest computed tomography (CT) revealed a cavitary lesion, suggesting a bronchopleural fistula with empyema. (B) Chest CT showed subsequent aspiration pneumonia in the right lower lobe. (C) Chest CT taken before the endobronchial procedure revealed the cavitary lesion (black arrow), suggesting a persistent bronchopleural fistula. (D) A CT image taken before the endobronchial procedure showed the fistula, in which the pleural cavity and bronchial tree communicated (black arrow).

The surgical procedure was successfully conducted as planned. The pathological diagnosis was minimally invasive adenocarcinoma, pT1miN0M0-IA1, according to the eighth edition of the TNM classification for lung cancer.^[7]^ Although prolonged air leakage had been seen postoperatively, the patient recovered and was discharged on postoperative day 14. However, on postoperative day 24, he complained of shaking chills with high fever and returned to our hospital. At admission, physical examination showed a blood pressure of 90/60 mm Hg, a heart rate of 120 bpm, and a body temperature of 40°C. Chest CT revealed BPF with resultant pneumonia, lung abscess, and empyema, which led to sepsis (Fig. 2A and B). Although the patient underwent partial resection of the infected right lung and closure of the fistula, air leakage persisted and his clinical condition deteriorated. On day 30 after the first surgery, he underwent open window thoracostomy (OWT) to improve the infection. After control of the infection had been achieved, on post-OWT days 80 and 91, he underwent surgical closure of the fistula and thoracoplasty with muscle transposition to close the OWT. The fistula was identified at the B6a stump in the operative field. Persistent air leakage remained after failure of thoracoplasty with OWT to control the air leak and sepsis. The fistula was difficult to detect bronchoscopically because the patient had undergone several surgical operations and his bronchi were deformed.

**Figure 3:**
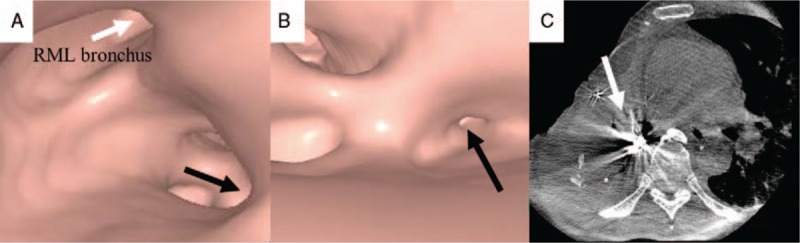
(A, B) Virtual bronchoscopic navigation images constructed on a SYNAPSE VINCENT indicated a bronchial route (black arrow) to the cavitary lesion. The image also showed the right middle lobe bronchus [white arrow in (A)]. (C) Computed tomography fluoroscopy confirmed that the tip of the catheter had been accurately inserted into the correct location (white arrow).

Chest CT images taken before the endoscopic procedure revealed a cavitary lesion communicating with a bronchial tree of the right lower lobe, which appeared to be the affected lesion (Fig. 2C and D). VBN images were constructed from CT images using a computer-assisted system (SYNAPSE VINCENT; Fujifilm Medical, Tokyo, Japan) (Fig. 3A and B).

**Figure 4:**
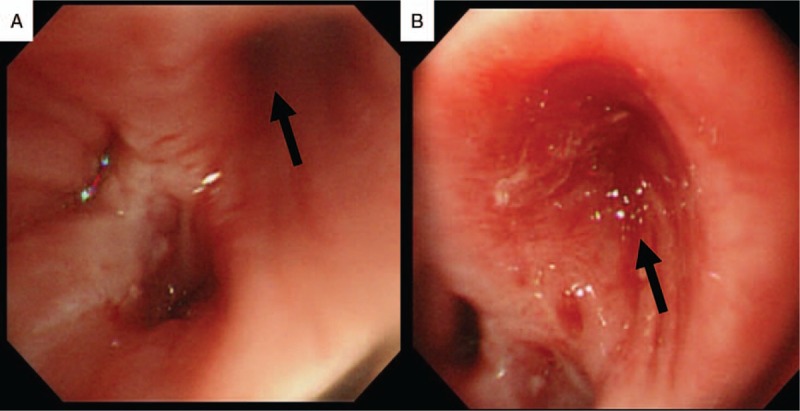
Bronchial occlusion with N-butyl cyanoacrylate glue. (A) The target lesion (black arrow) before the procedure. (B) The target lesion (black arrow) after the procedure.

On day 26 after the final operation, endoscopic bronchial occlusion was performed using a bronchoscope (BF-type 260; Olympus, Tokyo, Japan). Because the fistula appeared to be smaller than 5 mm, we decided to conduct endoscopic gluing, which has a high success rate in small fistulas.^[8,9]^ N-butyl cyanoacrylate (NBCA) was selected as a sealing glue because the strong bond between the NBCA glue and the affected tissue induces a reactive inflammatory response with permanent occlusion of the fistula even if the tissue glue embolus is blown out.^[8–10]^ Before the procedure, the patient consented to the use of NBCA glue in an attempt to remedy the fistula. Because the use of VBN navigation and CT fluoroscopy was apparently safe and the patient consented to the method, ethical approval was unnecessary. NBCA was mixed with lipiodol in a 1 : 3 ratio, and 1 mL of the liquid glue was drawn up in a syringe. Under mild sedation with intravenous administration of midazolam, a metal-tip catheter (PW-6C-1; Olympus) was inserted through the working channel of the bronchoscope into the target lesion using VBN guidance (Fig. 3A and B). The tip of the catheter was confirmed to have accurately reached the target lesion by CT fluoroscopy (Fig. 3C). NBCA mixed with lipiodol was then deployed in the affected lesion under CT fluoroscopy guidance (Fig. 4). The same endobronchial procedure was repeated 1 week later.

The endoscopic bronchial occlusion greatly reduced the air leakage. Before the procedure, a digital drainage system (Thopaz; Medela, Baar, Switzerland) for monitoring of air leaks showed 300 mL/min air flow. After the bronchial occlusion, the air flow from the leak remarkably decreased to <30 mL/min. The air leakage gradually ceased, and the chest drainage tubes were removed 2 weeks after the second endobronchial occlusion. No adverse events related to the endobronchial procedure occurred. Finally, the patient was discharged on day 22 after the final endoscopic procedure. No lung cancer or BPF recurred for 18 months postoperatively.

## 3 Discussion

BPF is a potentially fatal complication associated with a high mortality rate after pulmonary resection.^[2]^ The incidence of BPF ranges from 0.5% to 3.0% after lobectomy.^[3,11]^ BPF can be complicated by subsequent pneumonia, empyema, adult respiratory distress syndrome, and death.^[12]^ Thus, patients with postoperative BPF are often seen in an advanced state of sepsis due to pneumonia or empyema. Indeed, our patient was in a critical clinical condition on readmission.

BPF could have been avoided if we had performed surgical resection using a different technique. In hindsight, concomitant S6a subsegmentectomy would have caused the BPF. If we had conducted right upper lobectomy and concomitant wide wedge resection of the right lower lobe, which would not have required management of the anomalous B6a, the patient may not have developed BPF. This case study sheds light on the importance of the extent of resection in the presence of an anatomical anomaly.

Successful therapeutic modalities for empyema with BPF are OWT and thoracoplasty.^[2]^ Despite these aggressive surgical interventions, some patients require repeat surgery.^[1]^ Unfortunately, fatal outcomes may occur.^[1,13]^ In the present case, we conducted 4 surgical operations to treat the BPF. Although we were able to prevent the patient from developing a life-threatening condition, air leakage persisted after the surgical procedures. Completion right lower lobectomy or pneumonectomy could have been options for the treatment of BPF. However, completion lobectomy would have been extremely challenging due to extensive adhesion after several procedures. Moreover, we avoided completion pneumonectomy because it would have resulted in severe respiratory failure, although the BPF might have been cured earlier. Further surgery would not have rescued this patient. Therefore, we performed an endobronchial intervention.

Endobronchial occlusion is reportedly a useful technique with which to eliminate or reduce air leaks.^[14]^ Moreover, previous reports have suggested that endobronchial treatment can be an optimal alternative treatment for postoperative BPF.^[2,9,15]^ According to reports published after 2000, the overall success rate of endoscopic treatment ranges from 70% to 100%.^[8,9,15–17]^

NBCA was remarkably useful for curing the postoperative BPF in the present case. As reported previously, many bronchoscopic attempts to treat BPF have been described and include mechanical abrasion, vascular coils, fibrin or tissue glue, spigots, stents, silver nitrate, autologous endobronchial blood patches, and endobronchial valves.^[2,6,9,15–19]^ In our case, because the fistula seemed to be relatively small, we did not use silicon spigots or vascular coils, which are likely to be effective for larger fistulae.^[9]^ Moreover, we did not apply mechanical abrasion or submucosal injection because we feared that those procedures might deteriorate the fistula. NBCA, which has been widely used for endoscopic gluing, was selected in the procedure.^[8–10,20]^ NBCA glue injected into the fistula induces a local inflammatory response that causes proliferation of the bronchial mucosa. This process ensures long-term closure of the fistula.^[9]^ The procedure is easily performed through the fiberoptic bronchoscope. Endobronchial airway valves, which have recently been reported to be effective not only for lung volume reduction in emphysema but also for postoperative BPF or persistent air leakage, may have also been appropriate for the procedure in this case. Successful placement of the valves leads to resolution of BPF by eliciting segmental atelectasis, which in turn decreases airflow and allows adequate healing time.^[18]^ Although little data are available, endobronchial valves seem to be effective regardless of the size of the fistula.^[18]^ However, we adopted the NBCA gluing method because of its availability.

Although the endobronchial intervention was effective in closing the fistula in our case, previous reports have described difficulties in identifying the affected bronchi or the fistula.^[3,14]^ Even balloon occlusion tests can fail to detect the affected lesion in more than half of cases.^[14]^ In our case, we were unable to identify the fistula by conventional bronchoscopy.

CT fluoroscopy and VBN greatly contributed to the endobronchial occlusion in this case. Both techniques support transbronchial lung biopsy.^[4,5,21]^ Virtual bronchoscopy is also reportedly useful in the diagnosis of BPF.^[3]^ Virtual bronchoscopy may be an optimal diagnostic modality for BPF because it is a noninvasive study that simulates the endobronchial 3-dimensional view of the airway. Moreover, the successful use of VBN for endobronchial procedures has been previously described.^[6]^ CT fluoroscopy also played an important role in this case because it was necessary to confirm the location of the catheter. Notably, the NBCA used in this case could not be applied in the normal bronchi. If we had injected the NBCA glue into the normal bronchi, the patient would have developed respiratory problems. Identification of the precise location of the catheter was indispensable. Thus, CT fluoroscopy was useful for confirmation of the catheter's location before application of the NBCA glue.

Although endobronchial occlusion under CT fluoroscopy and VBN is a safe and effective procedure, it has some practical problems. First, a computer-based VBN system may have a high additional cost. However, a recent study showed that VBN images constructed from a CT workstation were useful for the diagnosis of small peripheral pulmonary nodules.^[22]^ Thus, no additional cost is required, and VBN images would be available in many hospitals. Second, exposure to radiation potentially limits the routine use of CT fluoroscopy for endobronchial interventions. To overcome this problem, we should carefully consider the indications for the use of CT fluoroscopy. In the present case, we required CT fluoroscopy guidance because the target lesion could not be missed. We conducted the endoscopic procedure twice. CT fluoroscopy was not used in the second endobronchial procedure because we confirmed the location of the fistula during the first procedure and attempted to protect the patient from radiation exposure.

In conclusion, CT fluoroscopy and VBN can be useful techniques for endobronchial fistula closure in patients with postoperative persistent BPF. To ensure the safety and efficacy of the endobronchial intervention, VBN images should be made before the procedure, and the catheter's location should be confirmed by CT fluoroscopy.

## Acknowledgment

We thank Angela Morben, DVM, ELS, from Edanz Group (www.edanzediting.com/ac) for editing a draft of this manuscript.
